# Fetomaternal outcome in patients with placenta previa

**DOI:** 10.12669/pjms.36.5.1647

**Published:** 2020

**Authors:** Tayyiba Wasim, Natasha Bushra, Saher Riaz, Hafiza Iqra Iqbal

**Affiliations:** 1 Dr. Tayyiba Wasim, FCPS, Department of Gynecology, Services Institute of Medical Sciences, Lahore, Pakistan; 2 Dr. Natasha, FCPS, Department of Gynecology, Services Institute of Medical Sciences, Lahore, Pakistan; 3 Dr. Saher Riaz, FCPS-I, Department of Gynecology, Services Institute of Medical Sciences, Lahore, Pakistan; 4 Dr. Hafiza Iqra Iqbal, FCPS-I, Department of Gynecology, Services Institute of Medical Sciences, Lahore, Pakistan

**Keywords:** Morbidly adherent placenta, Placenta previa, Postpartum hemorrhage caesarean hysterectomy

## Abstract

**Objectives:**

To assess maternal and fetal morbidity associated with placenta previa and morbidly adherent placenta (MAP).

**Methods:**

All patients with placenta previa who delivered in services hospital from April 1, 2017 to March 31, 2019 were included. Maternal and fetal outcomes were compared amongst patients with placenta previa and MAP.

**Results:**

Total of 8002 patients delivered with 152 (1.9%) diagnosed as placenta previa and 56 (36.8%) amongst them had MAP. One hundred thirty-one out of One hundred fifty-two (86.1%) of our patients were booked. Increased number of caesarean section, multi parity and anterior placenta had significant association with MAP (p<0.0001). Maternal morbidity in terms of postpartum hemorrhage >2000ml, caesarean hysterectomy, number of blood transfusions, bladder injury, need for ICU admission was significantly more in patients with MAP (p<0.0001). Case fatality was 3% with two maternal deaths in MAP and none in placenta previa. Fetal outcome was good in both groups as gestational age at delivery was 36 weeks or more, birth weight was ≥ 2.5 kg and >6 APGAR score (p<0.05). Two neonatal deaths occurred in MAP and one in placenta previa owing to prematurity.

**Conclusion:**

MAP is a dreadful complication of placenta previa with increased maternal morbidity. Regular antenatal care with adequate arrangement of blood transfusion and multidisciplinary approach can reduce maternal mortality.

## INTRODUCTION

Placenta previa complicates 0.3-1.5% of the pregnancies and it may lead to significant maternal morbidity and even death. It is also associated with poor neonatal outcome includes preterm delivery, low birth weight and perinatal death.^1^ Placenta previa invading the uterine wall becomes morbidly adherent placenta(MAP) in form of placenta accrete, increta and percreta. MAP can result in life threatening hemorrhage, disseminated intravascular coagulation and death.^2^,^3^

The rising trend of caesarean section has led to dramatic increase in incidence of placenta Previa and MAP in last few decades.^4^,^5^ Ultrasound has good diagnostic accuracy in diagnosis of placenta previa but in some patients, MAP is diagnosed intraoperatively and hence has catastrophic outcomes.^6^

Pakistan is fifth most populous country with maternal mortality of 178/100,000. Obstetric hemorrhage is major contributor of these maternal deaths with placenta previa along with MAP now being the major culprits.^7^,^8^ Morbidity with placenta previa and MAP can significantly be reduced if diagnosed antenatally. This will ensure arrangement in properly equipped hospital with multidisciplinary approach and availability of blood transfusion, anesthesia, ICU and neonatal facilities. This is extremely challenging in low resource countries where blood transfusion and operative services are not available at periphery where most of the population is residing. Repeated multiple studies which emphasize the underlying cause of ante and postpartum hemorrhage will go a long way in sensitizing people at government level to improve facility at primary, secondary and tertiary level.

We aimed to carry out this study to find frequency of placenta previa and MAP, along with maternal and fetal outcome. Our is the first large study from Pakistan to compare these outcomes amongst patients with placenta previa and MAP.

## METHODS

This cross sectional study was conducted at gynecology department, Services Institute of Medical Sciences/ Services Hospital Lahore, which is a tertiary care hospital attached to public sector medical college and receives booked and referred patients from April 1, 2017 to March 31, 2019. After getting ethical approval form ethical committee (IRB/315/SIMS) dated March 6, 2019, all patients with alive pregnancies after 24 weeks with placenta previa and MAP diagnosed on ultrasound or intra operatively whether booked or unbooked and underwent caesarean section in our department were included. Placenta previa was labelled as placenta partially or completely covering the os. All other were categorized as low lying placenta and were excluded from the study. MAP included placenta accreta, increta and percreta. We do not have MRI facility at hospital and it is expensive in private setup so ultrasound was the only modality used for diagnosis.

Baseline demographics including maternal age, gravidity, parity, education, booking status, previous obstetric outcome was recorded. The patients who had early booking were counselled about complications, need for blood arrangement, prolonged hospital stay and operative delivery. All those who had bleeding episodes or lived far away were admitted in hospital, rest were managed on OPD basis. Steroid injections were given at 30-32 weeks. Elective delivery was planned at 37 weeks. Preterm delivery was planned if patient had significant antepartum hemorrhage.

Preoperatively, arrangement of four units of blood for patients with placenta previa and six units of blood and six fresh frozen plasmas for patients with MAP was made. High risk consent including consent for hysterectomy was taken from all patients with MAP. They were operated by multidisciplinary team comprising of senior obstetrician, urologist, hematologist, anesthesiologist and ICU specialist. We do not have interventional radiology in our department so conservative management in terms of compression sutures, Uterine artery ligation and myometrial resection were employed for patients desiring fertility. All the rest had caesarean hysterectomy. Patients and babies were followed up till seven days after delivery.

Maternal outcomes of number of bleeding episodes, postpartum hemorrhage >2000ml, caesarean hysterectomy, number of blood transfusions, bladder injury, need for ICU admission, reopening and mortality were recorded. Fetal outcome was recorded as gestational age at delivery, birth weight, APGAR score, NICU admission and perinatal death. All data was compared amongst patients with MAP and those having placenta previa without abnormal adherence. Data was entered and analysed using SPSS version 23. The comparison between qualitative variables was done by using chi square test or fisher Exact test where appropriate. All P-values were two tailed and p-value of 0.05 or less was considered significant.

## RESULTS

Total number of 8002 patients delivered with 152 patients having placenta previa during the study period, giving a frequency of 1.9%. Fifty-six (36.8%) patients out of 152 had MAP. The patients having MAP were older and had high parity than those with placenta previa. 66.07% of patients were in age group 31-40 years while 52.08% of patients with placenta previa were less than 30 years (p<0.05). Majority (57.14%) of patients with MAP had high parity with only 21.87% of patients with placenta Previa had more than 3 children (p<0.0001). Gestational age was more than 34 weeks in both groups; 73.2% and 65.2% (p=0.332). Only 24 patients did not have a previous section, all the rest had a history of caesarean section. Increased number of caesarean section and anterior placenta previa were significantly associated with MAP (p=0.0001) Table-I.

**Table-I:** Demographic features of patients in placenta previa and MAP.

Demographic Profile	Morbidly Adherent Placenta		Non Adherent Placenta Previa		P-Value

	N=56	%	N=96	%	
***Age***					
18 -30 yrs	19	33.92%	50	52.08%	0.030
31 – 40 yrs	37	66.07%	46	47.91%	
***Education***					
Illiterate-Matric	15	26.78%	57	34.37%	0.418
FA-Graduation	41	73.21%	39	65.62%	
***Booking Status***					
Yes	50	89.2%	81	81.37%	0.332
No	6	10.71%	15	15.62%	
***Parity***					
≤3	24	42.85%	75	78.12%	0.397
>3	32	57.14%	21	21.87%	
***Past Obs History***					
Normal delivery	2	3.57%	22	22.91%	0.0001
1-2 C.Section	26	46.42 %	68	70.83%	
≥3 C.Section	28	50%	6	6.25%	
***Placental Position***					
Anterior	52	92.85%	57	59.37%	0.0001
Posterior	4	7.14%	39	40.62%	

Regarding maternal outcome (Table-II), episodes of antepartum hemorrhage was seen in 76.78% and 63.54% patients respectively in both groups (p>0.05). Patients with MAP had significantly more postpartum hemorrhage, blood transfusions, caesarean hysterectomy and need of intensive care unit (ICU) as compared to patients with placenta previa (p<0.0001). Seven (12.5%) patients with MAP had urinary bladder injury while two patients of MAP had to be reopened due to intraperitoneal bleed and both expired. Case fatality was 3%. None of the patients with placenta previa expired.

**Table-II:** Comparison of maternal outcome in placenta previa and MAP

Maternaal Outcome	Morbidly Adherent Placenta		Non Adherent Placenta Previa		P-Value

	N=56	%	N=96	%	
***Antenatal bleeding***					
No	13	23.21%	35	36.45%	0.090
Yes	43	76.78%	61	63.54%	
***PPH***					
<2000 ml	03	5.35%	73	76.04%	0.0001
>2000 ml	53	94.64%	23	23.95%	
***Blood transfusion***					
≤ 5 transfusions	18	32.14%	88	91.66%	0.0001
>5 transfusions	38	67.85%	8	8.33%	
***Bladder Injury***					
Yes	7	12.5%	0		0.0001
No	49	87.5%	96	100%	
***C Hysterectomy***					
Yes	44	78.57%	03	3.12%	0.0001
No	12	21.42%	93	96.87%	
***ICU Admission***					
Yes	19	33.92%	02	2.08%	0.0001
No	37	66.07%	94	97.91%	
***Re-Open Surgery***					
Yes	2	3.57%	00	0%	0.062
No	54	96.42%	96	100%	
***Mortality***					
Yes	02	3.57%	00	0%	0.062
No	54	96.42%	96	100%	

Majority of babies were born with birth weight ≥ 2.5 kg ((73.3% and 72.9%), 78.5% and 76.16% in both groups had good APGAR score(p=0.657). Only 8 (14.28%) babies of patients with MAP and 11 (11.45%) babies with placenta previa needed admission in Neonatal intensive care unit (NICU). Two babies of patients with MAP and 1 of placenta previa died due to prematurity (Table-III).

**Table-III:** Comparison of fetal outcome in placenta previa and MAP

Fetal Outcome	Morbidly Adherent Placenta		Non Adherent Placenta Previa		P-Value

	N=56	%	N=96	%	
***Baby Weight***					
<2.5 kg	15	26.7%	26	27.03%	0.657
2.5kg	41	73.3%	70	72.97%	
***Apgar Score***					
<6/10	12	21.42%	24	25%	0.645
≥6/10	44	78.57%	72	76.16%	
***Admission in NICU***					
Yes	08	14.28%	11	11.45%	0.611
No	48	85.71%	85	88.54%	
***Perinatal Death***					
Yes	2	3.57%	1	1.04%	0.279
No	54	96.42%	95	98.95%	

## DISCUSSION

The frequency of placenta previa in our study was 1.9% as compared to worldwide prevalence reported from 0.2-4.84%.^3^,^9^ Low prevalence has been reported from European and Sub Saharan African countries while high prevalence from Asian Studies but ethnicity could not be established as sole factor.^10^ The prevalence of MAP has risen in last few decades with incidence increasing from 1 in 30,000 pregnancies in 1960s to 1:533.^11^ This has been attributed to increase in caesarean section rate worldwide.^3^-^5^,^12^ This is also shown in our study where 96.4% of patients with MAP had prior caesarean section and 50% had more than three sections. Serious efforts should be made to curtail caesarean section at all levels as young patients with MAP not only suffer severe morbidity in terms of severe postpartum hemorrhage, massive blood transfusions, DIC, urinary bladder injury but also suffer from loss of fertility due to hysterectomy at very young age. MAP now accounts for 47% of peripartum hysterectomies.^13^ Position of placenta is an important determinant of adverse pregnancy outcome. Our study showed that 92.85% of patients with MAP and 59.37% patients with placenta previa had anterior placenta. This is related to presence of an anterior scar from previous section. Anterior location of placenta previa is an independent risk factor for increase chances of haemorrhage and caesarean hysterectomy.^14^

In our study, 86.1% patients were booked with regular antenatal care and hence had a chance to get correct diagnosis made on ultrasound preoperatively resulting in appropriate planning. Pagani G et al. in meta-analysis conclude that ultrasound if done properly, has good diagnostic accuracy with 90% sensitivity and 98% specificity for recognizing placental invasion.^6^

Ante and postpartum haemorrhage, caesarean hysterectomy and preterm birth are main morbidities in cases of placenta previa when compared to normally sited placenta.^9^,^15^,^16^ In our study, we compared the same morbidity outcomes in placenta previa and MAP which showed that patients with MAP had severe maternal morbidity as compared to placenta previa in terms of bad severe obstetric haemorrhage ( 94.6% vs 76.4%), massive blood transfusion(67.85% vs 8.33%), caesarean hysterectomy(78.5% vs3.12%),ICU admission(33.5% vs 2.08) and bladder injury(12.5% vs 0%) p<0.0001. Similar morbidity is reported from all studies worldwide.^2^-^5^,^11^-^14^,^17^-^19^

We had low case fatality of 3% as only two patients died. Antenatal care played an important part in improved outcome of these patients as 86.1% of our patients were booked. It allowed timely arrangement of blood and involvement of multidisciplinary team and low case fatality. Similar results with high morbidity and low mortality have been reported in a study from Lahore but number of patients was less.^18^ High maternal mortality has been reported from Tanzania and India mainly due to lack of antenatal care.^9^,^20^

Fetal outcome was good in patients with MAP and placenta previa in our study with majority(73.2% vs 65.6%) delivering after 36 weeks, with good APGAR and babies having birth weight ≥2.5kg and only three neonatal deaths due to prematurity(p>0.05). Some studies have reported adverse neonatal outcomes in these patients in terms of lower birth weight and preterm birth^3^-^5^,^16^ while others have reported term deliveries with good perinatal outcome.^11^,^21^ The reason may be difference in comparison groups and booking status of the patients. Antenatal care enables bed rest, steroid cover and blood transfusions in case of antepartum haemorrhage and helps in reduction of morbidity related to prematurity.

### Strength and Limitation of the study

The strength of our study was that it had large number of booked patients, otherwise studies from public sector hospitals have mostly emergency patients. Secondly, to our knowledge It is the largest study from Pakistan comparing outcomes in patients with placenta previa and MAP. It had the limitation of single tertiary centre study.

## CONCLUSION

Morbidly adherent placenta has emerged as significant complication of placenta previa owing to increased number of caesarean section. MAP has high maternal morbidity but mortality can be reduced by regular antenatal care and multidisciplinary approach. All efforts should be made to reduce caesarean sections and awareness should be created to deliver patients with MAP in properly equipped hospital.

### Author`s Contribution

**TW** conceptualized and designed the study, reviewed the manuscript and approved the final version. She is responsible for accuracy and integrity of work.

**NB** contributed to maintenance of data base and initial writing of script.

**SR and HII** did the data entry and statistical analysis.
